# Environmental Behaviors of *Bacillus thuringiensis* (*Bt*) Insecticidal Proteins and Their Effects on Microbial Ecology

**DOI:** 10.3390/plants11091212

**Published:** 2022-04-29

**Authors:** Yujie Li, Cui Wang, Lei Ge, Cong Hu, Guogan Wu, Yu Sun, Lili Song, Xiao Wu, Aihu Pan, Qinqing Xu, Jialiang Shi, Jingang Liang, Peng Li

**Affiliations:** 1 College of Food Sciences and Technology, Shanghai Ocean University, Shanghai 201306, China; lyj1187571462@163.com; 2 Biotechnology Research Institute, Shanghai Academy of Agricultural Sciences, Shanghai 201106, China; cuiwang87@126.com (C.W.); gl13084233857@163.com (L.G.); hucong819@163.com (C.H.); wgghappy@126.com (G.W.); yvessuen@hotmail.com (Y.S.); songlili@saas.sh.cn (L.S.); gwuxiao@126.com (X.W.); aihup@163.com (A.P.); 3 Shanghai Key Laboratory of Agricultural Genetics and Breeding, Shanghai 201106, China; 4 Shandong County Agricultural Technology Extension Center, Jinan 250003, China; xuqinqing1982@163.com; 5 Dezhou Academy of Agricultural Sciences, Dezhou 253000, China; shijialiang2004@163.com; 6 Development Center of Science and Technology, Ministry of Agriculture and Rural Affairs, Beijing 100176, China; 7 Shanghai Co-Elite Agricultural Sci-Tech (Group) Co., Ltd., Shanghai 201106, China

**Keywords:** *Bt* insecticide protein, environmental behaviors, *Bt* crops, *Bt* biopesticides, soil microorganisms

## Abstract

*Bt* proteins are crystal proteins produced by *Bacillus thuringiensis* (*Bt*) in the early stage of spore formation that exhibit highly specific insecticidal activities. The application of *Bt* proteins primarily includes *Bt* transgenic plants and *Bt* biopesticides. Transgenic crops with insect resistance (via *Bt*)/herbicide tolerance comprise the largest global area of agricultural planting. After artificial modification, *Bt* insecticidal proteins expressed from *Bt* can be released into soils through root exudates, pollen, and plant residues. In addition, the construction of *Bt* recombinant engineered strains through genetic engineering has become a major focus of *Bt* biopesticides, and the expressed *Bt* proteins will also remain in soil environments. *Bt* proteins expressed and released by *Bt* transgenic plants and *Bt* recombinant strains are structurally and functionally quite different from *Bt* prototoxins naturally expressed by *B. thuringiensis* in soils. The former can thus be regarded as an environmentally exogenous substance with insecticidal toxicity that may have potential ecological risks. Consequently, biosafety evaluations must be conducted before field tests and production of *Bt* plants or recombinant strains. This review summarizes the adsorption, retention, and degradation behavior of *Bt* insecticidal proteins in soils, in addition to their impacts on soil physical and chemical properties along with soil microbial diversity. The review provides a scientific framework for evaluating the environmental biosafety of *Bt* transgenic plants, *Bt* transgenic microorganisms, and their expression products. In addition, prospective research targets, research methods, and evaluation methods are highlighted based on current research of *Bt* proteins.

## 1. Introduction

*Bt* protein is a δ-endotoxin insecticidal crystal protein derived from the *Bt* protoxin produced by *Bacillus thuringiensis* (Bt) in the early stage of endospore formation. The crystal protein exhibits highly specific insecticidal activity due to protease hydrolysis [1]. Indeed, *Bt* exhibits poisoning effects on Lepidoptera, Diptera, Coleoptera, Hymenoptera, Hemiptera, Orthoptera, Mallophaga, and other insects, in addition to snails, nematodes, and protozoa [2,3,4]. Importantly, *Bt* proteins are selective for insects, but they are harmless to humans, vertebrates, and plants. Consequently, *Bt* accounts for about 90% of the production and use of biopesticides and has become the most widely used microbially produced pesticide globally, with widespread use in agriculture, forestry, fruit trees, vegetables, and environmental sanitation pest control [5,6]. However, *Bt* insecticides feature disadvantages, including narrow insecticidal spectra, poor stability, short residual effect periods, and the generation of insect resistance, which all limit their wider application.

The artificial design and optimization of *Bt* insecticidal proteins have led to the construction of high-efficiency and broad-spectrum recombinant insecticidal proteins, with recombinant engineered *Bt* strains being an effective means to solve the above problems [7]. For example, Wang et al. introduced the Cry3Aa7 gene encoding a coleopteran-specific insecticide into the wild *B. thuringiensis* strain G03 using electroporation, with the insecticide exhibiting high toxicity to lepidopteran pests [8]. The resultant recombinant engineered strain (G033A) was the first instance of a transgenic *Bt* engineered strain that was approved and registered as a pesticide in China [9]. In addition, *Bt* insecticidal proteins are also widely used in *Bt* transgenic crops that use genetic engineering technology to introduce exogenous beneficial genes into crop genomes. These introduced genes can be stably inherited, resulting in crops that have better agronomic traits and economic value [10,11]. 

**Figure 1:**
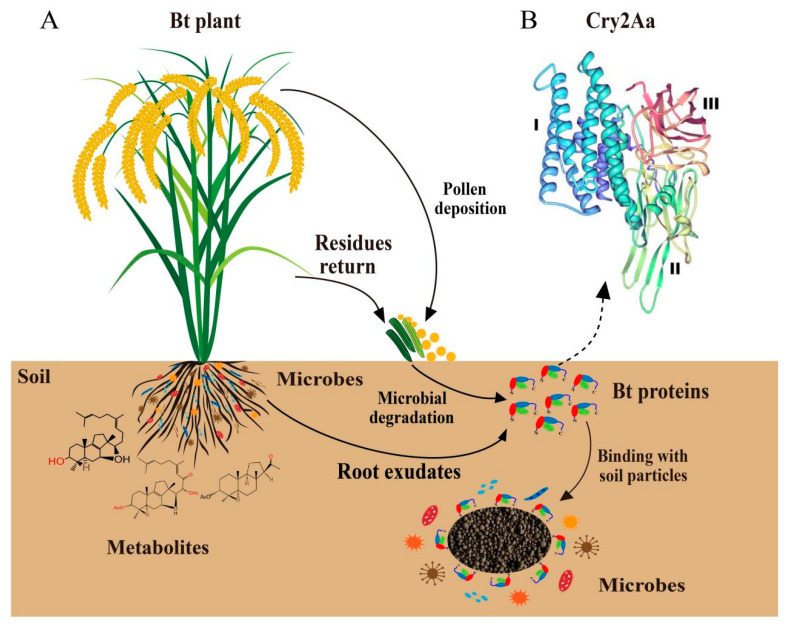
Environmental behaviors of *Bt* protein (**A**) and its three-dimensional structures (**B**). I, II, and III: domains I, II, and III.

The global large-scale commercial cultivation of genetically modified crops beginning in 1996 has led to the expansion of planted areas with each passing year, reaching 190.4 million hectares in 2019 (a ~112-fold increase compared to 1996). Among these, insect resistance (*Bt* gene)/herbicide tolerance composite traits within genetically modified crops comprise the largest global area of planted crops. In particular, transgenic insect-resistant cotton is a genetically modified *Bt* crop that has been approved for commercial cultivation in China [12]. *Bt* is the most widely used insect resistance gene and exhibits the greatest potential and application prospects for plant genetic engineering research. The *Bt* gene has been successfully introduced into tobacco [13], corn [14], and cotton [15] plants, among others, leading to numerous transgenic plant varieties or germplasm resources with good insect resistance. Rui et al. observed that the content of the *Bt* toxin secreted by rhizospheres can reach 200–300 ppb [16], while Valldor et al. reported that the Cry1Ab protein contents released by *Bt* transgenic maize Mon810 through root systems into soils reached 165 g/hectare [17]. Residual accumulated *Bt* proteins in the environment exceed the consumption by insect larvae and degradation by environmental factors, leading to potential impacts on the abundances, community structures, and functions of natural soil microbial communities (Figure 1A) [18,19]. Crecchio and Stotzky observed that the *Bt* proteins secreted by *Bt* maize roots can intimately associate with clay minerals and humic acids, among other soil substrates, leading to retained insecticidal activity in soils. These complex forms are more difficult to biodegrade compared with free state Bt, also suggesting the potential for sustained ecological risks [20].

In addition to the development of *Bt* recombinant engineered strains with higher insecticidal activity and wider insecticidal spectrum to delay insect resistance evolution, researchers have also commonly artificially modified the codons or promoter sequences of *Bt* to promote the expression of *Bt* in transgenic plants [21]. Consequently, the *Bt* proteins expressed by *Bt* transgenic plants are quite different from the *Bt* prototoxins expressed by the original *Bt* bacteria in terms of protein structure and function. Thus, *Bt* proteins that are expressed by *Bt* recombinant engineered strains and *Bt* transgenic plants represent exogenous environmental substances with insecticidal toxicity [22,23]. In addition, because *Bt* proteins can be complex with clays and humic acids when entering soils, they are not easily degraded by microorganisms and retain their biological activity for extended periods of time (Figure 1A). Consequently, the environmental behavior of exogenous *Bt* proteins in soil ecosystems and their impact on soil biodiversity should be systematically evaluated [24]. To improve the safety of genetically modified organisms and their products, China has implemented a safety evaluation system for transgenic plants (including *Bt* transgenic plants) and transgenic microorganisms (including those used in plant crops) [25]. Only transgenic products that have been evaluated for safety can now be planted in fields and commercially produced at a large scale. This paper summarizes the structure and mechanism of *Bt* insecticidal proteins, in addition to the retention, adsorption, and degradation of *Bt* insecticidal proteins in soils and their effects on soil physical and chemical properties along with soil microbial diversity. Moreover, we highlight prospects for potential *Bt* research targets in the future, enabling a scientific framework for the environmental and ecological security evaluation of *Bt* transgenic plants, *Bt* transgenic microorganisms, and their expression products. 

## 2. Expression and Mechanism of *Bt* Insecticidal Proteins

### 2.1. Formation and Structure of *Bt* Insecticidal Proteins

Crystals are formed at the same time as endospore formation by the *Bt* bacterium. The main components of these crystals are proteins with insecticidal activity, known as ICPs or δ-endotoxins. *Bt* proteins exhibit various forms, including bipyramidal (Cry1), cuboidal (Cry2), flat rectangle (Cry3A), irregular (Cry3B), spherical (Cry4A and Cry4B), bar-shape (Cry11A), amorphous, and mosaic morphologies (https://www.bpprc.org/, 27 April 2022) [26,27]. The shape, structure, and size of the *Bt* proteins are closely related to their insecticidal virulence and specificity [28,29]. Using transmission electron microscopy, Liu and Guo observed that the *Bt* strain SFZZ03 conducts cell division in addition to the formation and development of sporulation and parasporal crystals at different fermentation times. Most of the crystals produced by strain SFZZ-03 were blunt diamonds, although square and long rhombic forms were also produced, in addition to a few hexagonal, triangular, oval, and irregular polygons. Regardless of the crystal forms, mosaic shapes were widespread and may be related to the regulation of synthetic crystals produced by the *Bt* SFZZ-03 strain [30]. Li et al. investigated the relationship between different crystal morphologies and their insecticidal activities. Their results revealed that crystal shapes among different strains and crystal compositions were not identical, with different forms of crystals exhibiting different virulence characteristics against insects. For example, insecticidal crystal proteins that are highly virulent to Lepidoptera larvae are generally in the form of bipyramidal crystals [31]. In addition, *Bt* can produce vegetative insecticidal proteins (Vips) that show no homology with ICPs. Because of their novel spectrum of activity and lack of cross-resistance with ICPs, Vips have been considered second-generation insecticidal proteins. As the primary group of vip genes, vip3 genes encode proteins of about 88.5 kDa. Vip3 toxins also play an important role in killing a broad spectrum of Lepidoptera larvae [3]. Meanwhile, Vip3A transgenic crops have been developed, e.g., corn MIR162 and cotton COT102, which should be valuable for resistance management [32,33].

*Bt* crystal proteins contain three different domains: Domain I, Domain II, and Domain III; (Figure 1B) [34]. Domain I (the pore-forming domain) is located at the N-terminal of the Cry active protein and consists of a bundle of seven antiparallel α-helices. The domain is cleaved by proteolytic enzymes during toxin activation and is associated with toxins entering cell membranes and pore formation [35]. Domain II (the central domain) comprises three antiparallel β-folds and is a receptor recognition and binding site that determines the specificity of insecticidal protein targets [36]. Domain III (the galactose-binding domain) comprises two antiparallel β-folds and is related to receptor binding and pore formation [35]. Schnepf et al. identified that the active core region of the prototoxin activated by midgut protease (Domains I, II, and III) contained five conserved amino acid sequences, with three conserved regions outside of the active core [27]. In addition, the homology analysis of the amino acid sequences of different *Bt* proteins revealed that the amino acid sequence homology of Cry1Aa and Cry3Aa was about 36%, while the three-dimensional structures of Cry1Aa and Cry3Aa exhibit some similarities. In contrast, the homology of amino acid sequences in Cyt2Aa, Cry1Aa, and Cry3Aa is less than 20%. Cyt2Aa has a peculiar structural form, with its protein three-dimensional structure comprising a domain consisting of two outer α-helices wrapped around a mixed β-fold [37]. Cao et al. discovered that the Cry78Aa protein exhibits high insecticidal activity against both *Laodelphax striatellus Fallén* (Hemiptera: Delphacidae) and *Nilaparvata lugens* *Stál* (Hemiptera: Delphacidae). The Cry78Aa crystal structure comprises two independent domains, including a β-trefoil domain at the N-terminal similar to the S-type Ricin B lectin, while a β-pore forming domain of the aerolysin family is present at the C-terminal. The insecticidal activity of Cry78Aa depends on the synergistic action of these two domains [38]. 

Given the large area of *Bt* transgenic crop application and the widespread use of *Bt* insecticides, many insect populations have developed resistance to *Bt* insecticidal crystal proteins, albeit to varying degrees [39,40]. Consequently, identifying new *Bt* insecticidal proteins with high insecticidal activity and wide insecticidal spectra are important research foci globally. Further, the establishment of a new method for effectively gene mining and identifying *Bt* insecticidal proteins is critical. For example, a method combining PCR restriction fragment length polymorphism (PCR-RFLP) and single-oligonucleotide nested-PCR has been used for such purposes. Using this method, a novel *Cry* gene (*Cry30Fa1*) was isolated and identified that encodes 687 amino acid residues and exhibits a molecular weight of 77.1 kDa, while carrying significant insecticidal effects against *Plutella xylostella* *L*. (Lepidoptera: Plutellidae) and *Aedes aegypti L.* (Diptera: Culicidae) [41]. Liu et al. re-designed the previous tool to provide a novel, high-throughput, and local software BtToxin_Digger, which can be directly used to handle large-scale genomic and metagenomic data to predict all kinds of putative toxin genes. It is more suitable for large-scale toxin gene mining, and at the same time, it can easily implement the high-throughput analysis [42]. The system was validated by using 21 *Bt* strains from the laboratory. Among them, three potentially represent novel *cry* gene types (primary ranks) and five of them became *cry* holotypes [43].

### 2.2. Mechanism of Action for *Bt* Insecticidal Proteins

Mechanistic investigations of *Bt* insecticidal proteins have primarily focused on lepidopteran insects [36] and evaluated three different models, namely, the perforated classic model, the signaling pathway model, and the continuous binding model [44]. Bravo et al. proposed the classical model to explain the mechanism of *Bt* insecticidal crystal protein using the Cry1Ab protein as the focus. In this model, *Bt* prototoxin is ingested by insects and is then solubilized in the alkaline intestinal environment, followed by hydrolysis via insect midgut proteases, leading to the production of low molecular proteins (~60 kDa) with insecticidal activity [45]. These low molecular weight *Bt* proteins can bind to the specific receptor Aminopeptidase N (APN), Alkaline phosphatase (ALP), and Cadherin (CAD), forming polymeric structures. The polymer then penetrates the lipid membrane, causing membrane perforation and disrupting the osmotic pressure balance within the insect cells, leading to cellular dehydration and death [35,45]. In the signaling pathway model, the *Bt* insecticidal protein activates an Mg^2+^-dependent signal transduction system and protein kinase A (PKA) through specific binding to CAD. The induction of the adenylate cyclase (AC)/protein kinase A (PKA) signaling pathway then initiates a series of cytological events that include membrane blebbing, the appearance of nuclear ghosts, and cell swelling that are followed by cell lysis, and ultimately cell death [46]. In the continuous binding model, *Bt* prototoxin becomes a low molecular protein after activation by midgut protease. The protein then binds to CAD analogs and undergoes conformational changes, including the hydrolyzed oligomer structure of the domain, followed by binding to secondary receptors like Aminopeptidase that can promote the insertion of the toxin into cell membranes, ultimately leading to cell apoptosis and insect death [5,47]. The perforated classic model has been most invoked in studies of *Bt* proteins. Nevertheless, many questions regarding the model require resolution, including how do insect cell membranes form perforated channels and how do monomers and polymers exert toxic effects and alter the strength of toxicity? 

The signaling pathway model has not gained widespread acceptance due to its lack of scientific demonstration. Further, it is unclear how the anterior and posterior processes in the continuous binding model are associated with each other, while the specific insecticidal mechanism also remains unclear [48]. Notably, numerous proteases are present in the midgut cavities of insects. The compositions of different proteases will directly affect the degradation and activation activities of *Bt* proteins, thereby playing a critical role in the specificity of insecticides. Moreover, the active site of the Cry protein is the midgut epithelial cells of insects. The possibility of different Cry insecticidal proteins binding the same receptor protein in the same insect requires further investigation [49].

Wang et al. developed the perforated classic model and discovered a novel “double channel” mechanism for *Bt* insecticidal protein activity against *Hübner* (Lepidoptera: Noctuidae), while also first identifying a pair of highly structurally similar and functionally overlapping *Bt* receptors, ABCC2 and ABCC3. After binding and interacting with *Bt* insecticidal proteins, the receptors form permeability pores on the membrane of the midgut, leading to damage and shedding of midgut cells that then causes larvae to stop feeding and die [50]. Sun et al. studied the contribution of two paralogous ATP-binding cassette (ABC) transporters and two APN to *Bt* Cry1Ac toxicity in the diamondback moth, *P. xylostella*, using CRISPR/Cas9 to generate a series of homozygous polygenic knockout strains. The results showed that a double-gene knockout strain, in which the two paralogous ABC transporters ABCC2 and ABCC3 were deleted, exhibited 4482-fold resistance to the Cry1A toxin, and a double-gene knockout strain in which APN1 and APN3a were deleted exhibited 1425-fold resistance to the Cry1Ac toxin. Furthermore, genetic crosses of the two double-gene knockouts yielded a hybrid strain in which all four receptor genes were deleted, and this resulted in a >34,000-fold resistance, indicating that while both types of receptors need to be present for the toxin to be fully effective, there is a level of functional redundancy between them [51]. Guo et al. elaborated the mechanism of *Bt* Cry toxin resistance in *P. xylostella*, and they confirmed that the MAPK-mediated differential expression of APN and other midgut genes did indeed lead to Cry1Ac resistance in *P. xylostella* [52]. In further study, they carried out a genome-wide characterization of all of the MAPK orthologs in *P. xylostella* to define their phylogenetic relationships and confirm their evolutionary conserved modules. The results from quantitative phosphoproteomic analyses, combined with functional validation studies using specific inhibitors and dsRNAs, lead them to establish a MAPK “road map”, where p38 and ERK MAPK signaling pathways, in large part, mount a resistance response against *Bt* toxins through regulating the differential expression of multiple Cry toxin receptors and their non-receptor paralogs in the *P. xylostella* midgut [53]. Batool et al. and Zhao et al. analyzed the regulatory mechanism of *Bt* proteins through the C-type lectin CTLGA9 and ATP-binding protein of *A. eegypti.* These proteins may compete with *Bt* proteins to bind ALP1 and APN receptors, thereby inhibiting their insecticidal toxicity. These results could provide new insights into an in-depth understanding of the mechanism of *Bt* [54,55]. Gao et al. used eukaryotic and prokaryotic expression systems to express the cadherin gene (HaCad and PxCad) and other extracellular fragments (HaCad-TBR and PxCad-TBR) of *H. armigera* and *P. xylostella* in Sf9 cells and *Escherichia coli*, respectively. The authors also analyzed their binding characteristics with different *Bt* toxins, cytotoxicity mediation, and synergistic effects with toxins. The results suggested that PxCad may be the functional receptor of the Cry1Ac toxin, although the binding affinity for Cry1Ac toxin and cytotoxicity mediation are significantly weaker than for HaCad. Both cadherins can bind to some of the toxins Cry2A, Cry1B, Cry1C, and Cry1F, but they are not functional receptors for the toxins. Notably, although PxCad is not a functional receptor for the Cry1F toxin, the PxCad-TBR fragment exhibits a synergistic effect with both Cry1Ac and Cry1F toxin against *P. xylostella* [56]. 

Xie et al. designed four novel genetically engineered antibodies (GEAbs), wherein GEAb-dV_L_ comprises two light chains incorporating overlapping binding sites with Cry1A and Cry1B proteins. The GEAb also exhibited a high binding affinity with Brush border membrane vesicles (BBMVs) in *P. xylostella* midguts. GEAb-dV_L_ is structurally different from the Cry toxin in that it does not have the α-helix perforation structure and exhibits weak binding activity against CAD and other Cry toxin receptors. Nevertheless, GEAb-dV_L_ can still stably bind *P. xylostella* BBMVs, resulting in midgut cell damage and insect death. Interestingly, GEAb-dV_L_ exhibits a different insecticidal mechanism compared to the Cry toxin perforation pattern, providing an important contrast for the development of next-generation biological control pesticide products and key insect-resistance genes [57]. 

## 3. Environmental Fate of *Bt* Insecticidal Proteins in Soils

### 3.1. Adsorption, Retention, and Degradation of *Bt* Insecticidal Proteins in Soils

After *Bt* proteins are released into soils, they closely bind cohesive soil and humus particles by adsorption, where they can remain for a long time (Figure 1A). Consequently, the potential environmental safety of *Bt* proteins is a concern worthy of widespread attention [58]. Tapp and Stotzky first proposed that active particles in soils exhibit adsorption effects on *Bt* insecticidal proteins, wherein different soil types have different adsorption effects on *Bt* proteins. Cohesive particles in the soil can rapidly adsorb >70% *Bt* protein within 30 min. During adsorption, the concentration of *Bt* protein first increases, followed by a later decrease, ultimately achieving equilibrium within 5–6 h [59]. Stotzky and Crecchio observed that the adsorption of soil surface-active particles can effectively inhibit degradation by soil microorganisms. Soil clays, humic acids, and montmorillonite–humic acid–polymeric aluminum hydroxide complexes closely bind *Bt* proteins, preventing *Bt* protein from being used as a carbon source by microorganisms. Indeed, microbial growth in media containing *Bt* protein was significantly reduced by the above complexes compared with growth in the presence of free *Bt* proteins [60]. 

Conde and Patino observed that the interactions between *Bt* proteins and the surfaces of soil particles are maintained by physical and chemical processes, including cation exchange, electrostatic interactions, hydrophobic forces, hydrogen bonding, and van der Waals forces [61]. To better understand the mechanism of *Bt* protein adsorption by different soil types, Helassa et al. investigated the adsorption and degradation of purified Cry1Aa proteins in sandy and clay soils. Decreased Cry1Aa protein concentrations were not significantly correlated with soil microbial degradation, but rather to physicochemical interactions that occurred on soil surfaces, wherein hydrophobic interactions may play an important role in determining the interactions of Cry1Aa proteins with surfaces [62]. Wang also analyzed the adsorption of the *Bt* protein using five types of soils as substrates, revealing that the adsorption capacity of the active soil particles was negatively correlated with soil pH. These conclusions are consistent with those of Tapp, wherein the adsorption capacity of montmorillonite is inversely proportional to the acidity and alkalinity of soils over a certain pH range, with higher soil pH leading to lesser adsorption of *Bt* proteins [22]. Further, Yao et al. identified a linear relationship between soil organic matter content and the soil adsorption capacity for *Bt* protein, wherein soil cohesive particles adsorbed *Bt* proteins, and the higher organic matter content in soil particles led to greater *Bt* protein adsorption [63]. The studies of Zhou et al. and She et al. both observed that the adsorption capacity for *Bt* proteins differed among various soil particles, with the adsorption capacity for *Bt* protein by goethite, kaolin, and silicon dioxide sequentially decreasing. Further, the insecticidal activity and anti-UV degradation ability of *Bt* were enhanced after protein adsorption by attapulgite [64,65]. Zhou et al. investigated the effects of *Bt* protein application on Pb(II) adsorption in Ultisols and Vertisols, observing that Pb(II) adsorption decreased in both soil types at *Bt* toxin concentrations from 0 to 10 mg/L. This dynamic may be related to competition for adsorption sites and the formation of Pb toxin complexes. The adsorption capacity of Pb(II) in Vertisols was higher than in Ultisols, while the influential trend of *Bt* toxins was the opposite of the maximum adsorption capacity of Pb(II) in the two soils. In addition, the adsorption capacity of Pb(II) decreased when *Bt* protein was applied in the Ultisols and Vertisols, thereby increasing the environmental risk of Pb(II) [66]. Consequently, these results suggest that close attention should be paid to the potential risk of *Bt* proteins in soil environments and functioning [58].

The degradation of *Bt* proteins has been widely studied in soils, and its degradation rate in soils is affected by the type and concentration of *Bt* protein, soil types, soil microbial community composition, and soil pH [67]. Wang et al. analyzed the soil degradation of the *Bt* protein released by four different *Bt* transgenic maize (34B24, NK58-D1, R×601RR/YG, and Nongda 61). At 25 °C, the degradation of *Bt* protein occurred over three stages, namely, the early release stage, the rapidly declining stage, and the low, stable stage for all four *Bt* maize straw types. A comparison of the degradation in sterile and non-sterile soils indicated that biotic decomposition was one of the main factors contributing to degradation [68]. Stotzky also determined the insecticidal activity of residual proteins in soils that were subjected to the application of exogenous Cry1Ab protein and planted with *Bt* transgenic maize, revealing the insecticidal activity of Cry1Ab can persist for 120–180 d. Further, *Bt* protein activity could still be detected after 1–2 years within *Bt* transgenic maize straw [69]. In contrast, Zhang et al. observed that *Bt* proteins rapidly decompose in soils and do not accumulate. The authors measured Cry1Ac protein content after *Bt* transgenic cotton straw was applied, revealing a lack of Cry1Ac protein activity in soils after 180 d, and that Cry1Ac proteins did not continuously accumulate in the field [70]. Feng et al. used a shift-log model to describe the degradation of Cry1Ab proteins under different environmental conditions, observing results consistent with those of Wang et al. [68]. They also evaluated the effects of soil temperature, water content, and pH on Cry1Ab protein degradation. Among these, soil temperature significantly affected the degradation of Cry1Ab, with the degradation rate accelerating at higher temperatures. In contrast, soil moisture content and pH had no significant effects on the degradation of Cry1Ab [71]. Deng et al. also found that temperature conditions can alter the soil microbial activity, thus further affecting the persistence of Cry toxins. When the ambient temperature is low and even reaches freezing point at night in winter, it will inhibit the microbial activity and delay the degradation process [72]. To verify the hypothesis that soil type might influence the degradation rate of *Bt* protein in soils, Zhou investigated the effects of soils from four different regions on the adsorption of *Bt* proteins in laboratory soil culture experiments. Soils from Zhengzhou and Gongzhuling exhibited similar degradation trends for soil *Bt* proteins, while soils from Beijing and Jinan exhibited similar later degradation of soil *Bt* proteins [73]. Thus, the adsorption and degradation of soil *Bt* proteins are closely related to soil types.

### 3.2. Transformation Fate of *Bt* Insecticidal Proteins in Soils

The proportion of free *Bt* proteins extracted from paddy soils using the commonly employed phosphate-buffered saline with Tween 20 (PBST) method was relatively low in one study, although the proportions of *Bt* proteins bound to soil particles were high, difficult to extract, and could not be determined by enzyme-linked immunosorbent assays (ELISAs) [74]. ELISA tests cannot currently distinguish the *Bt* proteins from exogenous *Bt* proteins released by transgenic plants compared to the *Bt* prototoxins produced by soil *Bt* bacteria. Consequently, the environmental behavior and biological effects of exogenous *Bt* proteins cannot be accurately evaluated at present. Consequently, rather than measuring the low content and free states of the *Bt* protein in soils to assess its accumulation and retention in soils, environmental safety assessments of exogenous *Bt* insecticidal proteins should focus on the transformation direction and ecological effects of *Bt* proteins that are expressed in *Bt* transgenic crops and *Bt* recombinant engineered strains in soil environments [17,74]. Valldor et al. investigated the fate of *Bt* insecticidal proteins in sandy soils and clays using Cry1Ab labeled with ^14^C radioisotopes. The ^14^CO_2_ production rate due to ^14^C-Cry1Ab mineralization decreased with soil cultivation time in the two soil types, with the ^14^C flux accounting for 15–40% of the total ^14^C flux. At 29 and 37 days of soil culture, 16–23% of the ^14^C flux entered into microbial carbon, suggesting that microorganisms can use Cry1Ab as a substrate for their growth. The proportion of ^14^C in Cry1Ab remaining in soil due to adsorption by soil particles was the highest (approximately 40–80%). Due to the high degradation efficiency of ^14^C-Cry1Ab in sandy soils, the *Bt* protein content was very low in clay based on ELISA detection [17]. In another study using ^14^C-labelled Cry protein, similar rates of mineralization were found for a Cry1Ac protein in loamy sand with a comparably low pH [75]. Thus, it is speculated that clay particle adsorption is the main factor limiting the microbial degradation of *Bt* proteins. *Bt* protein can be absorbed onto soil particles and humic acid to form bound *Bt* protein, leading to the retaining of insecticidal activity. Bound *Bt* protein is more difficult to biodegrade compared to free *Bt* protein and carries more potential ecological risks [20,76]. The desorption rate of *Bt* protein by soil particles and humic acid is affected by soil properties, agricultural practices, and other factors [77,78]. Consequently, these factors should be given more attention to understand the behavior and fate of exogenous *Bt* proteins in agricultural ecosystems, rather than using ELISA tests to estimate the retention time of water-soluble *Bt* proteins in soils.

## 4. Effects of *Bt* Insecticidal Proteins on Soil Microbial Ecology

### 4.1. Effects of *Bt* Insecticidal Proteins on Soil Physicochemical Properties

Soil is an important site for the exchange of matter and energy in terrestrial ecosystems. The life activities of soil organisms largely depend on the physicochemical properties of soils. Consequently, an important consideration is whether exogenous *Bt* protein in soils affects the physical and chemical properties of soils that could then affect nutrient transformation processes. Such considerations could represent important indicators for evaluating the safety of field-released *Bt* transgenic plants and *Bt* transgenic microorganisms [79]. Liu applied two insecticidal crystal proteins (Cry1Ab and Cry1Ac) to three soil types, including paddy soils originating from fluvo-aquic soil, red soils, and yellow-brown soils. The application of *Bt* proteins increased soil pH in the short term compared to control soils, while no significant differences were identified between the control and experimental soils after long-term cultivation. Further, *Bt* protein application decreased the degradation rate of organic matter in the paddy soils that originated from fluvo-aquic soils. Moreover, the addition of *Bt* protein increased the nitrate–nitrogen content of the red soils, wherein the amount of total nitrogen in the red soils was higher than in the control after cultivation over a long period of time. Lastly, the ratio of C/N in the yellow-brown soils was lower in the experimental soils compared to the control [79]. Chen et al. analyzed the changes in untargeted metabolomics for the soil metabolite profiling of transgenic and non-transgenic maize. As predicted, the metabolomic profile greatly differed between transgenic and non-transgenic maize cultivars at all stages, and the difference was more prominent in the middle stage. These results suggest that genetic modification with the *cry1Ah* gene-altered maize can alter soil metabolism [80]. Our group also analyzed the dynamic changes of the physicochemical properties associated with soil cultivation by applying different concentrations of *Bt* protein to paddy soils at different times. No significant differences were observed in the physicochemical indices when comparing soils with different concentrations of *Bt* protein and control soils without *Bt* protein in the early stages of soil cultivation (i.e., at 1, 5, and 10 d). In contrast, NH_4_^+^-N and NO_2_^−^-N were significantly higher at the later stages of soil cultivation (50 and 100 d) compared to control soils without *Bt* protein application, although no significant difference was observed for NO_3_^−^-N in soils when *Bt* was applied at 500 ng/g. The above results suggest that the evaluation of environmental effects of *Bt* proteins should consider the concentration of *Bt* protein and its persistence time in soils (unpublished data).

Few studies have evaluated the effects of *Bt* proteins on soil physicochemical properties, and those that have primarily focused on the effects of root exudates of *Bt* transgenic crops and from straw returned to soils. Chen et al. analyzed changes in the available nutrient content in the rhizosphere soils of four transgenic *Bt* and non-*Bt* cotton species at different growth stages. The nitrate–nitrogen content of transgenic *Bt* cotton was significantly different from conventional cotton at many life stages. Further, the content of ammonium–nitrogen was significantly different from conventional cotton in the squaring, flowering, and boll stages. In addition, the content of available phosphorus differed, with the Lumian No. 28 variety exhibiting significantly higher levels than conventional cotton in the squaring stage, and the Lumian No. 36 variety exhibiting significantly higher levels than conventional cotton at the seeding stage. Thus, the planting and growth period of transgenic *Bt* cotton were the primary factors affecting *Bt* protein residues and the content of available nutrients in rhizosphere soils [81]. To thoroughly investigate the effects of transgenic *Bt* crop straw returned to soils on soil physicochemical properties, Yu investigated the effects of rice straw that was returned to fields on the soil physicochemical properties, denitrifying enzymes, denitrifying rates, and microbial diversity. The experiments were conducted under flooded conditions and used two transgenic *Bt* rice and two non-transgenic rice straw materials. Compared with non-transgenic *Bt* rice, the transgenic *Bt* rice straw returned to the field promoted soil denitrification, but no significant differences were observed in total nitrogen, organic matter, and other nutrient content in the paddy soils [82]. Zhang et al. also analyzed four types of *Bt* cotton with different levels of insect resistance, in addition to one non-transgenic conventional cotton variety (Simian no. 3). After one or two years of planting, all of the cotton straws were mechanically crushed and returned to the field in situ, followed by the measurement of changes in soil nutrient contents. The levels of organic matter, available phosphorus, available nitrogen, available potassium, and total nitrogen remarkably increased in soils within both the first and second years of cotton planting, as did soil pH. However, significant differences were not observed in the variation of all nutrient contents between *Bt* transgenic treatments and non-transgenic treatments [83].

### 4.2. Effects of *Bt* Insecticidal Proteins on Soil Microbial Community Diversity

Soil microbial communities are the most active soil biota and participate in soil organic matter decomposition, humus synthesis, and nutrient transformation, and promote soil development and formation. Further, they are important biological indicators for evaluating soil health [84,85,86]. Exogenous *Bt* protein enters soils and rapidly binds clays and humic acids to form bound *Bt* protein, leading to significant changes in its chemical composition and structure that may then adversely affect non-target organisms [24]. Rui et al. observed that the application of the *Bt* protein did not significantly alter the number of phosphate-dissolving and potassium-dissolving bacteria in soils based on plate counting measurements. Further, the abundances of nitrogen-fixing bacteria significantly decreased when *Bt* toxin concentrations were 500 ng/g or higher. The highest concentration of *Bt* toxin in the rhizosphere of *Bt* cotton NuCOTN99^B^ was 300 ng/g. Thus, differences in the numbers of functional soil bacteria between *Bt* cotton and non-*Bt* cotton treatments did not result from *Bt* toxin toxicity [16]. Li et al. added one of the most commonly used *Bt* proteins in *Bt* crops, Cry1Ac, to soils and evaluated the changes in soil bacterial, fungal, and archaeal diversities and community structures using terminal restriction fragment length polymorphism (T-RFLP) and real-time PCR (qPCR). The application of Cry1Ac did not significantly change the community diversity of microorganisms. In contrast, other studies have reported that the application of exogenous *Bt* protein can significantly alter the structure of soil microbial communities [87]. For example, Liu applied two insecticidal crystal proteins (Cry1Ab and Cry1Ac) to three soil types using paddy soils originating from fluvo-aquic soil, red soils, and yellow-brown soils, and then analyzed the changes in soil microbial genetic diversity using denaturing gradient gel electrophoresis (DGGE). Cry1Ac application increased diversity based on the Shannon index in paddy soils that originated from fluvo-aquic soils. Further, the application of both Cry1Ab and Cry1Ac increased microbial diversity in the yellow-brown soils [79]. Guan et al. performed a two-year field experiment that featured the cultivation of three *Bt* transgenic oilseed rape (*Brassica napus* L.) plant lines to evaluate their effects on rhizosphere microorganisms. The Shannon’s diversity, richness, and evenness of the soil microbial community increased significantly in transgenic GT1 and GT5 lines during the flowering stage [88]. Frouz et al. conducted a three-year field and laboratory study on the effect of *Bt* corn on the soil microbial community and decomposition rates of corn post-harvest residues, and found that *Bt* corn may have a deleterious effect on decomposers in the laboratory; however, this effect was minor and restricted to the initial stages of decomposition, and was undetectable in long-term field experiments [89]. Hong et al. investigated the effect of planting insect-resistant and herbicide-tolerant transgenic maize on rhizospheric microbial communities, and the results showed that at the genus level, planting transgenic maize significantly decreased the relative abundance of rhizospheric *Candidatus Nitrososphaera* at the jointing and mature stages [90]. These studies suggest that *Bt* proteins can impact microbial community structures and microbial diversity present within soils. The inconsistent results of the above studies may be due to the varying experiments using different *Bt* protein types, soil types, soil incubation times, and the analytical methods used in the experiments.

Previously, our group evaluated the impact of transgenic *Bt* crop cultivation on microbial community diversity. Specifically, microbial community structures were analyzed in rhizosphere soils using DGGE at the seedling, tiller, booting, heading, and mature stages of *Bt* transgenic rice in comparison with non-transgenic control rice. *Bt* rice cultivation had little effect on the dominant rhizosphere bacterial, fungal, and actinobacterial communities [91]. In addition, we evaluated the responses of fungal population sizes and community compositions via 18S rRNA gene sequencing when growing the transgenic insect-resistant cotton SGK321 variety expressing *Cry1Ac/CpTI* proteins. The population sizes of rhizosphere fungi associated with SGK321 taproots at the seedling stage were significantly higher compared to within lateral roots, suggesting that root microhabitats were influential towards fungal communities. Further, no significant differences were observed for fungal community sizes when comparing the rhizospheres of SGK321 and SY321 from the same root zones, suggesting that fungal abundances were not affected by *Bt* protein production in the root tissues of transgenic *Bt* cotton [92]. Plate-count enumeration and high-throughput sequencing were used by Li et al. and Qi et al. to evaluate the abundances of soil microorganisms associated with the long-term cultivation of transgenic insect-resistant cotton expressing Cry1Ac. A lack of significant variation was observed for the number of bacteria, fungi, *Azotobacter*, denitrifying bacteria, ammonia-oxidizing bacteria, and diversity indices of microbial communities when comparing transgenic cotton expressing Cry1Ac and non-transgenic cotton [93,94]. Xu et al. employed stable isotope probing and high-throughput sequencing to identify the active microorganisms involved in *Bt*-containing straw decomposition. Their results suggested that *Bt* rice exerts a significant, but transient, impact on soil microorganisms during microbial straw decomposition [95]. Zuo et al. also observed that transgenic poplars did not affect the physical and chemical properties of soils or soil microbial community structures, although community structures were affected by location and season [96]. Zhang et al. found that the Shannon–Wiener index of soil microbial communities in transgenic *Bt* cotton fields was significantly higher compared to those of non-transgenic cotton fields 30 days after cotton straw was returned to the fields [70]. Wu et al. used the high-throughput sequencing of bacterial 16S rRNA genes to evaluate the effects on the bacterial community diversity in soils of *Bt* rice grown for three consecutive years. No significant changes in bacterial community structures were observed for rhizosphere soils between *Bt* rice and non-*Bt* rice soils, while planting time had a greater effect on rhizosphere soil bacterial community composition than did cultivar type [97]. Wang et al. explicitly investigated the effect of transgenic rice on its active rhizosphere microflora by combining high-throughput sequencing with the SIP technique. The high-throughput sequencing of the bacterial 16S rRNA gene showed that transgenic rice did not significantly change the soil bacterial community structure compared with its parental variety. The soil bacterial community structure of transgenic and parental-labeled microbes was not significantly different, but was significantly different from those of the non-parental varieties, indicating that planting transgenic *Bt* rice had a limited impact on the soil microbiome [98]. In a two-year-long trial, Fan et al. found that insect-resistant transgenic maize carrying the *cry1le* gene did not affect the soil fauna [99]. Zhang et al. employed PCR-DGGE to monitor the rhizosphere soil microbial communities after three years cultivation of NC 33B in northern China. The results showed that the population sizes and community structures of eubacteria, fungi, and actinomycetes in rhizosphere soil were markedly affected by natural variations in the environment related to cotton growth stages. However, there was no significant difference in the eubacterial, fungal, or actinomycete population size and community structures in rhizosphere soil between NC 33B and its non-transgenic parent [100]. Even where *Bt* transgenic rice had been planted for 8 years, soil enzymatic activities and microbial biomass were also observed with no consistently significant changes [101]. Chen et al. examined and compared the effects of a non-transgenic maize cultivar and an insect-resistant transgenic maize cultivar genetically engineered with *cry1Ah* gene from *Bt* on the rhizosphere bacterial community using 16S rDNA amplicon sequencing and soil metabolome profile, using UPLC/MS analysis at six different growth stages. They found no significant differences in bacterial community composition or diversity at any growth stage between the two cultivars. These studies suggest that *Bt* crops have no significant effects on the soil microbial community [80].

### 4.3. Effects of *Bt* Insecticidal Proteins on Functional Diversity of Soil Microorganisms

The functional diversity of soil microorganisms refers to the functions that soil microbial communities can perform and the results from these functions, including, for example, nutrient decomposition, nutrient transformation, and promoting or inhibiting plant growth. These functions carry important significance for soil ecological functions and natural elemental cycles [102,103]. Liu observed that the application of *Bt* proteins inhibited the activity of nitrate reductases in paddy soils originating from fluvo-aquic and yellow-brown soils. Further, application of *Bt* proteins inhibited the activity of nitrite reductases in three soil types, including paddy soils originating from fluvo-aquic soils, red soils, and yellow-brown soils [79]. Yaqoob et al. found a reduced rate of phosphate solubilization and auxin biosynthesis during the maturity stages of both years when compared with that of the early stages, although these had no harmful effect on the biochemical or molecular characteristics of isolated soil bacteria from *Bt* cotton rhizosphere [104]. The effects of transgenic *Bt* crops on the functional diversity of soil microorganisms have also been extensively reported. Wu et al. determined that returning transgenic *Bt* rice straw to fields altered the soil dehydrogenase activity [105]. Liu et al. analyzed the rhizosphere soils of transgenic *Bt* rice grown for two years and observed seasonal changes corresponding to significant differences in phosphatase activity, dehydrogenase activity, respiration, methanogenesis, and fungal community composition in rhizosphere soils, although these metrics were not significantly affected by transgenic *Bt* rice cultivation [106]. Our group observed that the activities of dehydrogenases, invertases, phenol oxidases, acid phosphatases, ureases, and proteases were not significantly different between the soils of *Bt* and non-*Bt* rice. In addition, a Biolog system was used to evaluate the effects of *Bt* rice on the functional diversity of microbial communities. Although differences were observed in carbon substrate utilization between *Bt* and non-*Bt* rice at the seedling, tillering, and heading stages, these differences were transient [91]. Zhang et al. observed that *Bt* transgenic cotton significantly improved the utilization of carbon sources such as amino acids, amines, and carbohydrates by soil microbial communities in comparison to non-transgenic cotton 30 days after returning the straw to the fields [70]. Liang et al. observed a lack of differences in the functional diversity of microbial communities and the utilization of carbon sources in rhizosphere soils between transgenic maize IE09S034 and non-transgenic maize soils after two consecutive years of field experiments [107]. Further, Luan collected rhizosphere soils at the seedling, flowering, and maturity stages of transgenic *Bt* maize over two consecutive years, observing that the planted transgenic insect-resistant maize did not significantly affect the functional diversity of the rhizosphere soil microbial communities [108]. Xin observed that different maize growth stages and experimental years affect the functional diversity of microbial communities, and this may be related to the year of transgenic crop planting, in addition to the growth and development stages [109]. Li et al. explored how the responses of soil enzymatic activity varied across *Bt* crops or in different growth periods. The results showed that the activities of dehydrogenase, β-glucosidase, urease, nitrate reductase, alkaline phosphatase, and arylsulfatase significantly increased under *Bt* crop cultivation with residues incorporation. Further, the response ratios of soil enzymes varied with *Bt* crop types and growth periods [110]. Escalas et al. reported that numerous factors influence the functional diversity of soil microbial communities, including soil properties, climate types, individual microbial metabolisms, and community sizes [103]. Therefore, long-term systematic monitoring studies are necessary for evaluating the effects of *Bt* insecticidal proteins on the functional diversity of soil microorganisms at large scales.

## 5. Perspectives

Investigating the environmental behavior and ecological effects of exogenous *Bt* insecticidal proteins in soils is extremely important for understanding changes in soil structures and physicochemical properties, in addition to changes in microbial communities and the responses of functional diversity during the degradation and transformation of *Bt* insecticidal proteins. Understanding these dynamics will provide a scientific framework and theoretical reference for evaluating the environmental safety of transgenic *Bt* plants and *Bt*-recombinant engineered strains. Future studies should focus on conducting research in the two critical areas described below. 

### 5.1. Evaluating Environmental Behaviors of *Bt* Insecticidal Proteins Using Stable Isotope Tracing

Current ELISA methods for measuring *Bt* protein content cannot distinguish between exogenous recombinant *Bt* proteins and *Bt* protoxins produced by soil *Bt* bacteria, and thus cannot accurately evaluate the environmental behaviors and the biological effects of exogenous *Bt* proteins [17]. Valldor et al. obtained ^14^C-labeled Cry1Ab from batch fermentations with recombinant *E. coli* using ^14^C-labeled glycerol as the cultivation carbon source [111]. Although radioisotopes feature high sensitivity and low production costs, they are hazardous to operators, thereby limiting their research applications [112]. Stable isotope-labeled peptides and proteins are used to investigate subjects using physical, chemical, and biological methods by evaluating their traces, retention sites, and abundances in study systems using mass spectrometry and emission spectroscopy. Consequently, the method is commonly used in the detection and tracking of biological samples [113]. In particular, stable isotope mass spectrometry can be used to trace and quantitatively analyze the transformation, distribution, and dynamics of *Bt* protein carbon and nitrogen components among different forms of carbon and nitrogen, while effectively circumventing the influence of original *Bt* proteins in soils. Many stable isotope-labeled small-molecule compounds are commercially produced, although the development of singly labeled *Bt* proteins using ^13^C and ^15^N stable isotopes has not been reported. Our group recently used M9 medium with glucose as the sole carbon source and ammonium chloride as the sole nitrogen source to produce ^13^C/^15^N singly labeled *Bt* proteins with high purity and insecticidal activity from natural biosynthesis pathways. The resultant description, entitled “A method of preparing Cry protein with stable isotope ^13^C labeling”, has been patented (202010227154. X) and provides experimental materials and technical support for evaluating the environmental safety and ecological effects of *Bt* proteins [50]. Nevertheless, it is unknown whether the structure and stability of the above stable isotope-labeled *Bt* proteins differ from natural *Bt* proteins, thus requiring further analysis. In addition, soil physicochemical properties are important factors that affect the adsorption and degradation of *Bt* insecticidal proteins. In subsequent experiments, it will be necessary to analyze the environmental behavioral differences of *Bt* proteins in different types of soil.

### 5.2. Analysis of Microbial Ecological Effects of *Bt* Insecticidal Proteins Using Microbiome Techniques

The selection of a suitable analytical method to comprehensively and accurately analyze microbial communities and functional diversity response mechanisms is critical for evaluating the degradation and transformation of *Bt* proteins in soil. Many microorganisms in natural environments cannot be cultivated under conventional experimental conditions, although the isolation and cultivation of pure microbial cultures remain the basis for studies of microbial physiology, functioning, and genetics. High-throughput sequencing technology based on microbiomics can illuminate the community structures of soil microorganisms at a broad level, while also resolving changes in the relative abundances of specific microorganisms involved in certain functions. Combined with changes in key soil elements, these techniques can reveal important molecular regulatory mechanisms of microbial physiological and ecological processes in soils [114]. Consequently, current studies of microorganisms in environments have advanced from relying on pure cultures of microorganisms to studying the metabolisms of microbial communities by combining pure cultures and high-throughput sequencing [115]. Our group has performed the macrogenomic analysis of the microbial community functions involved in the degradation of Cry1Ab/Ac proteins, revealing that *Thermobifida, Streptomyces, Achromobacter, Noviherbaspirillum,* and *Pseumdomonas* were significantly involved in these processes. We speculate that these microorganisms are involved in the degradation and carbonaceous transformation of Cry1Ab/Ac. Further, among these taxa, *Streptomyces, Achromobacter,* and *Pseumdomonas* can all produce protein hydrolytic enzymes that can catalyze the hydrolysis of peptides or proteins into amino acids. A strain of *Streptomyces griseus* was subsequently isolated to purity, and a single cultivation experiment was performed using Cry1Ab/Ac proteins as substrates, with initial results suggesting that *Streptomyces griseus* can catabolize and utilize Cry1Ab/Ac proteins (unpublished data). In addition, soil metabolites can be considered phenotypes or signatures of soil microbial community changes, because alterations experienced at the organism and enzyme level will manifest as modified metabolite profiles [116]. Resolving soil metabolomes will contribute to a better understanding of the soil microbial ecological effects of exogenous *Bt* proteins. Consequently, the combined analysis of microbiomics and metabolomics is an important direction for further research to comprehensively identify the correlations between soil microorganisms, metabolites, and exogenous *Bt* proteins from multiple perspectives and levels.

## Data Availability

Not applicable.
